# Asthma diagnosis and treatment – 1007. Clinical application of forced oscillation technique for children with asthma

**DOI:** 10.1186/1939-4551-6-S1-P7

**Published:** 2013-04-23

**Authors:** Keigo Kainuma, Mizuho Nagao, Mayumi Sugimoto, Takao Fujisawa

**Affiliations:** 1 Institute for Clinical Research, Mie National Hosipital, Japan

## Background

Forced oscillation technique (FOT) measures respiratory impedance by applying external pressures to the respiratory system and makes it possible to evaluate various aspect of airway physiology including small airways. MasterScreen IOS® (IOS) has been widely utilized as a FOT. Recently, a new FOT, Mostgraph®, has been available in Japan. Clinical experience including reference values of FOT, especially, for Japanese children, however have not been determined and correlations of the 2 FOTs , MostGraph® and IOS, are not known.

## Objectives

To establish clinical utility of 2 FOTs, IOS and Mostgraph, we aimed to determine reference values of FOT parameters by the 2 technique in Japanese children.

## Subjects and methods

We performed FOT by using IOS and Mostgraph in 825 volunteer children from 6 to 18 years of age. ISSAC questionnaire was used to identify asthma and other allergies. In addition, 345 children with asthma who were treated at Mie National Hospital were also enrolled and IOS measurements at 3593 occasions were analyzed on the basis of clinical status of asthma.

## Results

All the FOT parameters were strongly dependent on the height and we created regression equations corrected for height from the data obtained from 542 and 399 non-asthma subjects for IOS and Mostgraph, respectively. All the parameters from IOS and MostGraph were significantly correlated. However, correlation coefficients were relatively high in R5 and AX and low in X5 and Fres, with Spearmann’s correlation coefficients of 0.62, 0.58, 0.39, and 0.41, respectively. Resistance parameters of both the FOTs in asthma were significantly higher than those in non-asthma subjects and correlated with severity of asthma.

## Conclusions

Reference values of 2 methods of FOT for Japanese children were established. Because of the differences in oscillation generation methods, IOS and MostGraph may represents different aspect of airway physiology.

